# The association between prenatal cannabis use and congenital birth defects in offspring: A systematic review and meta-analysis

**DOI:** 10.1192/j.eurpsy.2024.184

**Published:** 2024-08-27

**Authors:** A. W. Tadesse, G. Ayano, B. A. Dachew, B. S. Tusa, Y. Damtie, K. Betts, R. Alati

**Affiliations:** ^1^School of Population Health, Curtin University, Perth, Australia; ^2^School of Population Health, Curtin University, Perth, Austria

## Abstract

**Introduction:**

A body of research has examined the association between prenatal cannabis use and congenital birth defects in offspring; however, these studies have not been synthesised. We performed a comprehensive synthesis of existing research to test whether there is an association between prenatal cannabis use and congenital birth defects in exposed offspring.

**Objectives:**

The aim of this study was to conduct a comprehensive systematic review and meta-analysis of existing evidence to synthesise the association between prenatal cannabis use and congenital birth defects in exposed offspring.

**Methods:**

In line with the preregistered protocol (PROSPERO: CRD42022368623), we systematically searched PubMed/Medline, CINHAL, EMBASE, Web of Science, ProQuest, Psych-Info, and Google Scholar for published articles until 4 April 2023. The methodological quality of the included studies was appraised by the Newcastle-Ottawa Quality Assessment Scale (NOS). A meta-analysis was carried out to report the pooled effect estimates from the included studies. We further performed subgroup, leave-one-out sensitivity, and meta-regression analyses, which increased the robustness of our findings.

**Results:**

Thirty observational studies (i.e., fifteen case-control and fifteen cohort studies) with 229,930 cases of birth defects and 26,826,741 controls (healthy babies) were included in the final analysis. We found that offspring exposed to maternal prenatal cannabis had a 56%, 69%, 47%, 23%, and 13% increased risk of any birth defects (irrespective of specific body system) [RR = 1.56: 95 % CI 1.28 – 1.92], defects of the gastrointestinal [RR = 1.69: 95 % CI 1.37 – 2.09], cardiovascular/heart [RR = 1.47: 95 % CI 1.09 – 1.97], central nervous systems [RR = 1.43: 95 % CI 1.09 – 1.89], and facial/oral cleft [RR = 1.13: 95 % CI 1.08 – 1.18], respectively.

**Conclusions:**

The findings from the current study suggest that maternal prenatal cannabis exposure is associated with a higher risk of birth defects in offspring. The findings highlight the importance of promotive and preventive strategies to reduce cannabis use during pregnancy that contribute to minimising the risk of birth defects in offspring.

**Disclosure of Interest:**

None Declared

